# Mapping Dural and Periosteal SV2C, a Botulinum Toxin A Receptor, in the Mouse

**DOI:** 10.3390/toxins17100509

**Published:** 2025-10-17

**Authors:** Anisa Dehghani, Agustin Melo-Carrillo, Andrew M. Strassman, Ron S. Broide, Aubrey Manack Adams, Brett Dabruzzo, Mitchell F. Brin, Rami Burstein

**Affiliations:** 1 Department of Anesthesia, Critical Care and Pain Medicine, Beth Israel Deaconess Medical Center, Boston, MA 02115, USA; 2 Department of Anaesthesia, Harvard Medical School, Boston, MA 02215, USA; 3 Allergan, an Abbvie Company, Irvine, CA 92612, USA; 4 Department of Neurology, University of California, Irvine, CA 92697, USA

**Keywords:** migraine, headache, pain, tension-type headache, muscle tenderness, trigeminovascular, botox, neurotoxin

## Abstract

Objectives: There has been a long-standing debate over the presence or absence of receptors for botulinum toxin A (BoNT/A) in cephalic areas relevant to migraine pathophysiology and onabotulinumtoxinA (onabotA) sites of action in migraine prevention. To address this issue, we sought to investigate for the first time whether synaptic vesicle protein 2C (SV2C), one member of the SV2 receptor family, is present in axons innervating the dura and periosteum. Methods: Single- and double- labeling immunohistochemical techniques were used to map and characterize the distribution of axons containing SV2C, the third isoform of the SV2 glycoprotein, in the mouse dura and periosteum. Results: Dense networks of axons containing SV2C receptors were distributed throughout all regions of the dura and periosteum. In the dura, SV2C-LIR axons were found in 43% of all peripherin-LIR fibers, 49% of all CGRP-LIR fibers, and 75% of all NaV1.8-LIR fibers. In the periosteum, SV2C-LIR was found in 38% of all peripherin-LIR fibers, 53% of all CGRP-LIR fibers, and 68% of all NaV1.8-LIR fibers. Conclusions: We interpret these findings as suggesting that many of the labeled axons are peripheral nerve axons (peripherin-positive) of unmyelinated sensory and possibly parasympathetic origin (CGRP-positive), and that some of these sensory axons are nociceptors (NaV1.8-positive). Clinically, these findings demonstrate an abundance of axons containing onabotA receptors in the vicinity of scalp structures commonly injected with onabotA for the treatment of chronic migraine. Dense labeling in the periosteum provides another rationale for the possibility that onabotA injections in this layer of the scalp may be advantageous.

## 1. Introduction

Botulinum neurotoxin type A (onabotA) is a neurotoxin capable of blocking neuroexocytosis, the release of neurotransmitters/neuropeptides from presynaptic axonal terminals, and the insertion of ion channels and receptors into synaptic membranes [1,2]. It achieves these inhibitory effects by cleaving the synaptosomal-associated protein of the 25 kDa molecular weight (SNAP-25) component of the soluble *N*-ethylmaleimide-sensitive factor attachment protein receptor (SNARE) complex [3,4], rendering it dysfunctional to the extent that it does allow the docking and possibly priming but impairs fusion of synaptic vesicles into the synaptic membrane [3].

For its ability to disrupt communication between nerve endings of efferent motor axons and the muscles they innervate, botulinum toxin A (BoNT/A) is commercially available in a number of formulations (eg., onabotA (BOTOX) and incobotulinumtoxinA (Xeomin)) to treat dystonia and spasm, two painful neurological conditions characterized by focal overactivity of somatic muscles [5,6,7,8].

In recent years, it has become evident that onabotA’s ability to prevent the fusion of synaptic vesicles is not unique to peripheral nerve endings of motor neurons. It also occurs in nerve endings of selective classes of sensory and autonomic fibers [9,10,11,12,13,14,15]. Consequently, the therapeutic use of onabotA began to expand, to the extent that it is now approved by the FDA for the treatment of chronic migraine [16,17].

For the treatment of migraine, where the pathophysiology of the headache itself is likely to involve intracranial meningeal nociceptors supplying the dura and its vasculature, as well as extracranial periosteal and pericranial muscles, onabotA is injected into multiple pericranial and neck muscles whose tendons are attached to the periosteum of all bones of the calvaria. While it is not fully understood how extracranial injections of onabotA can alter synaptic transmission or neuronal responses originating in activation of nerve endings in the intracranial meninges, preclinical data show that extracranial injections of onabotA can in fact reduce the responsiveness of unmyelinated meningeal nociceptors to stimulation of their intracranial dural receptive fields with TRPV1 and TRPA1 agonists [15] or cortical spreading depression (CSD) [18]. There are two recently described networks of axons that innervate both intra- as well as extracranial tissues such as the dura, periosteum, and pericranial muscles. One network originates in trigeminal ganglion neurons whose axons reach the dura (i.e., meningeal nociceptors) and issue collateral branches that cross the calvaria bones along suture lines to reach the periosteum, galea aponeurotica, and some pericranial muscles [19,20,21]. Another network originates in C2 dorsal root ganglia neurons whose axons course through neck muscles before crossing bones of the calvaria (from outside to inside) and terminating in the occipital dura overlying the cerebellum [22]. These two networks are thought to ‘allow’ extracranial onabotA injections to influence intracranial, durally driven synaptic transmission and neuronal responses.

Given the abundance of evidence for the involvement of sensory axons in the dura, periosteum, and pericranial muscles in the initiation of the headache phase of migraine [23,24,25] and the strength of evidence for the therapeutic impact of onabotA in chronic migraine [16,17], we sought to map the distribution of onabotA receptors in sensory axons innervating these tissues. OnabotA is known to bind to synaptic vesicle protein receptor 2, which includes the isotypes SV2A, SV2B, and SV2C [26]. To the best of our knowledge, no study has ever attempted to map the distribution of any of these SV2 isoforms in cranial tissues relevant to migraine pathophysiology. In the current study, we used immunohistochemical methods to map the distribution of axons expressing SV2C in mouse dura and periosteum. We selected the SV2C isoform for mapping because onabotA has a much stronger interaction with the luminal L4 loop of SV2C than with the corresponding loops of SV2A and SV2B [26,27]. To further delineate the nature of the axons containing SV2C, we used double-labeling techniques to investigate its presence in axons containing peripherin-, CGRP-, and NaV1.8-like immunoreactivity.

## 2. Results

### 2.1. Dura Mater

**Figure 1:**
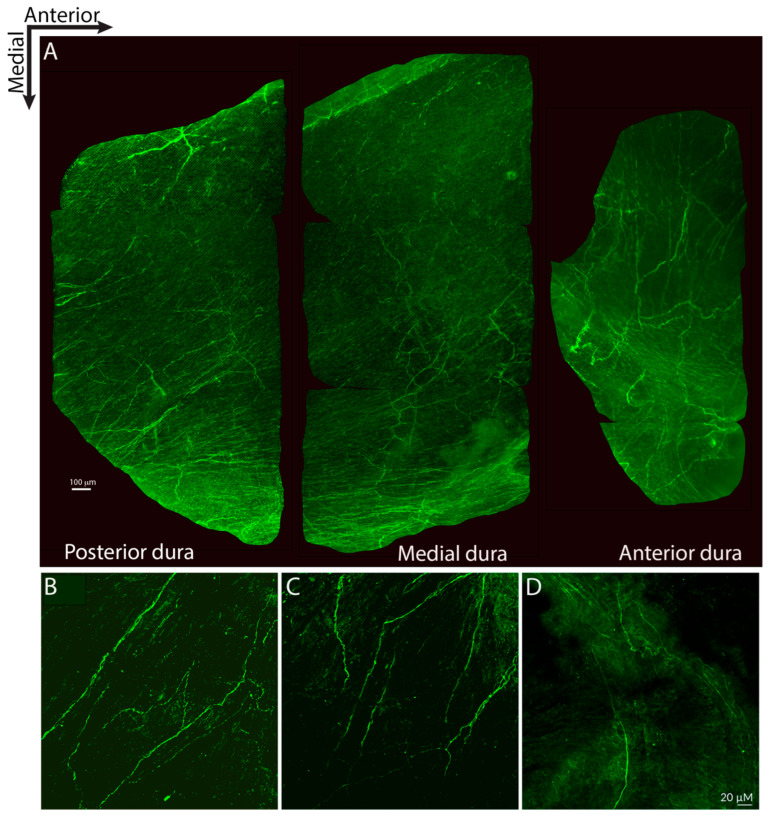
Distribution of SV2C-like immunoreactive (SV2C-LIR) axons of the dura. (**A**) Whole-mount of the dura overlying one hemisphere of the cortex, cut into three pieces that were arranged from posterior to anterior (anterior to the right, medial towards the bottom). Most axons were oriented anterior–posteriorly (**A**–**C**), while some were oriented mediolaterally (**A**,**D**) (10× and 20× objectives; confocal microscopy was used for all figures).

Distribution of SV2C-LIR fibers:

SV2C-positive fibers were distributed throughout all regions of the dura (Figure 1A). Most extended in an anterior–posterior orientation (Figure 1A–C), while some extended in a mediolateral orientation (Figure 1A,D). Some axons ran alongside dural blood vessels, and some ran perpendicularly across blood vessels.

**Figure 2:**
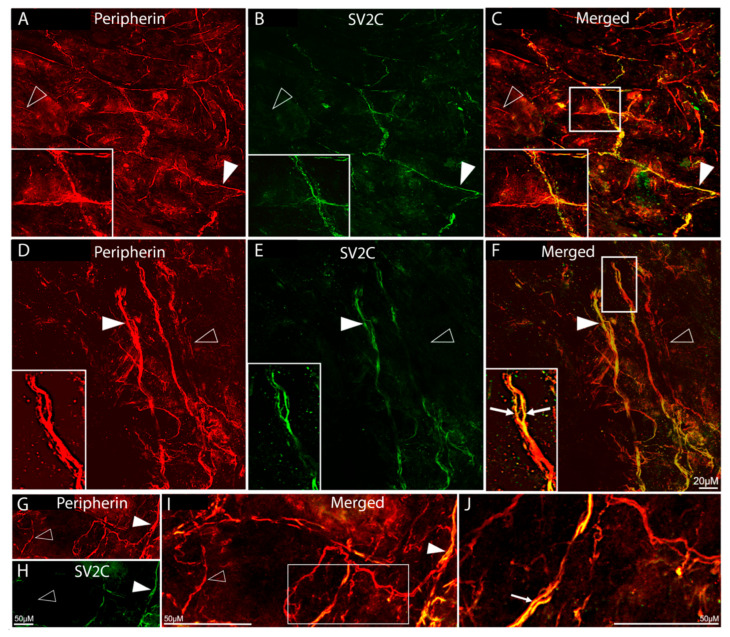
Colocalization of peripherin-LIR and SV2C-LIR in dural axons (20× objective). (**A**–**F**) Images of peripherin-LIR (red, **A**,**D**), SV2C-LIR (green, **B**,**E**), and co-localization of peripherin-LIR and SV2C-LIR (yellow, **C**,**F**) in dural axons. The rectangles in (**C**,**F**) mark areas that are enlarged in the insets in (**A**,**B**) and (**D**,**E**), respectively. (**G**–**I**) show images of peripherin-LIR (**G**), SV2C-LIR (**H**), and co-localization ((**I**); same microscope field as (**G**,**H**)). The rectangle in (**I**) is shown enlarged in (**J**). The filled arrowheads mark examples of axons that are double-labeled for SV2C and peripherin. The open arrowheads mark examples of axons that are single-labeled for peripherin-LIR only. The arrows in (**F**,**J**) mark bundles of peripherin-LIR axons that contain individual axons that co-localize peripherin-LIR andSV2C-LIR. Peripherin-like immunoreactivity confirms the neuronal origin of the labeled processes.

Distribution of SV2C/peripherin-LIR fibers (Figure 2):

Fibers showing peripherin-LIR were distributed throughout the dura and in all orientations (Figure 2A,D,G). Some were seen as large fiber bundles, some as individual axons within large bundles, and others as single axons. Of all peripherin-LIR fibers, 57% were single-labelled, expressing peripherin-LIR fibers only, and 43% were double-labelled, expressing both peripherin- and SV2C-LIR (Figure 2B,C,E,F,H–J). There was no SV2C-LIR fiber that was not also peripherin-LIR (Figure 2C,F,I,J), suggesting that in the dura, SV2C receptors are found in neuronal tissue only.

**Figure 3:**
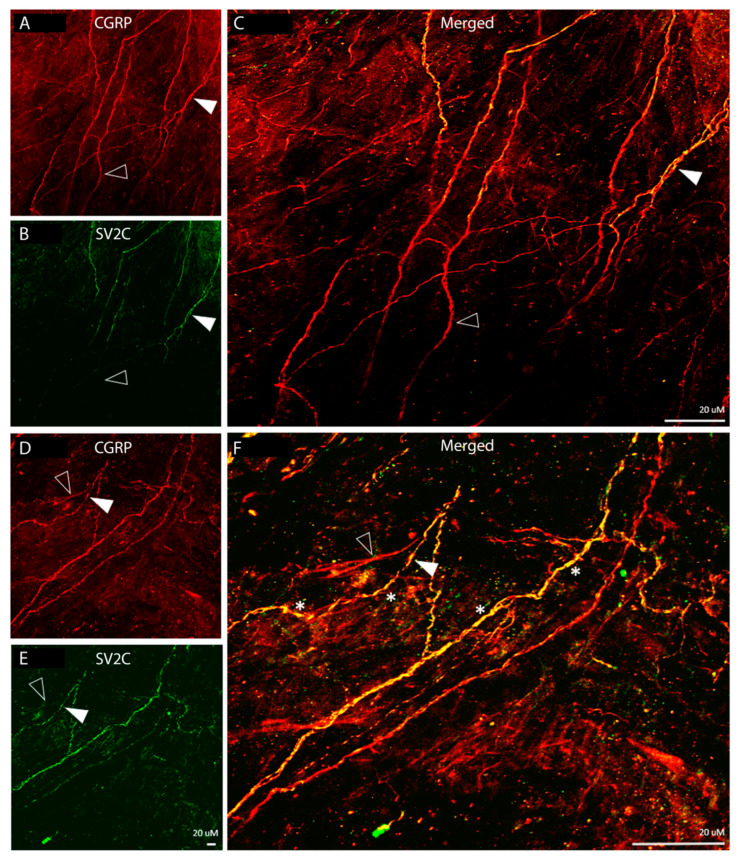
Colocalization of CGRP-LIR and SV2C-LIRin dural axons (20× objective). The images show CGRP-LIR (red, **A**,**D**), SV2C-LIR (green, **B**,**E**), and co-localization (yellow, **C**,**F**). The filled arrowheads depict double-labeled axons. The open arrowheads depict single-labeled CGRP-LIR axons. Note the presence of SV2C-LIR in CGRP-LIR axons (yellow, **C**,**F**), suggesting that some but not all C-fibers express SV2C receptors. Also note that some C-fibers axons express SV2C receptors in distinct areas only (* in (**F**)).

Distribution of SV2C/CGRP LIR fibers (Figure 3):

Fibers showing CGRP-LIR were distributed throughout the dura and in all orientations. Some were fiber bundles, some were single axons within bundles, and only a few were detected as individual axons (Figure 3A,D). Of all the CGRP-LIR fibers, 51% were single-labelled, expressing CGRP-LIR fibers only, and 49% were double-labelled, expressing both CGRP- and SV2C-LIR (Figure 3B,C,E,F). There was no SV2C-LIR fiber that was not also CGRP-LIR (Figure 3C,F). Of note, SV2C-LIR was present in an even, continuous distribution in some fibers but exhibited a discontinuous, patchy distribution in others (* in Figure 3F), suggesting that some axons express SV2C receptors throughout whereas others may express these receptors in distinct parts only.

**Figure 4:**
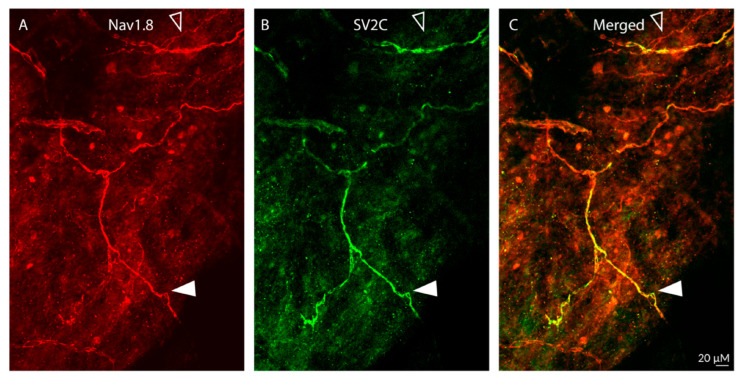
Colocalization of Nav1.8-LIR and SV2C-LIR in dural axons (20× objective). The images show Nav1.8-LIR (**A**), SV2C-LIR, (**B**), and co-localization (**C**, yellow) in dural axons. The filled arrowheads depict a double-labeled axon. The open arrowheads depict a single-labeled Nav1.8-LIR axon. Note presence of SV2C-LIR in some Nav1.8-LIR axons, suggesting the presence of SV2C receptors in some but not all sensory C-fibers.

Distribution of SV2C/NaV1.8 LIR fibers (Figure 4):

As expected, the distribution of fibers containing NaV1.8-LIR was far less dense than the distribution of peripherin- or CGRP-LIR fibers (Figure 4A). Fibers showing Nav1.8-LIR were also seen in bundles containing non-NaV1.8-LIR axons (Figure 4A). Of all Nav1.8-LIR fibers, only 25% were single-labelled, expressing NaV1.8-LIR only. In contrast, the majority (75%) of NaV1.8-LIR fibers were also SV2C-LIR (Figure 4B). There was no SV2C-LIR fiber that was not also NaV1.8-LIR (Figure 4C), suggesting that some but not all unmyelinated C-class meningeal nociceptors express SV2C receptors.

### 2.2. Calvarial Periosteum

**Figure 5:**
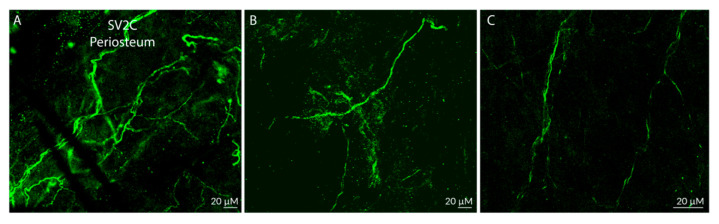
SV2C-LIRe axons in the periosteum. (**A**–**C**) show examples of axon bundles or single axons in periosteal tissue from three different animals. Note that most axons (**A**,**B**) are oriented anterior–posteriorly (lower left corner to upper right corner) and some are oriented mediolaterally (**C**), similar to the dura (20× objective).

Distribution of SV2C-LIR fibers (Figure 5):

SV2C-LIR fibers were distributed throughout the periosteum (below the galea aponeurotica). Their density resembled the density of SV2C-LIR in the dura. Unlike in the dura, where many SV2C-LIR fibers were seen as individual axons, in the periosteum they were seen mainly within large fiber bundles and, to a lesser extent, as single axons (Figure 5A–C).

**Figure 6:**
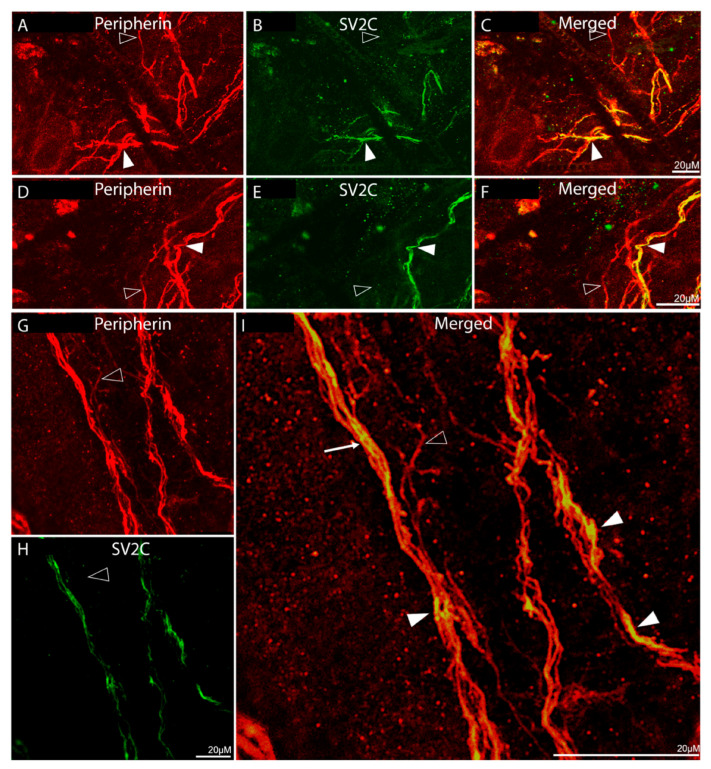
Colocalization of peripherin-LIR and SV2C-LIR in axons of the periosteum (20× objective). (**A**–**F**) Images of peripherin-LIR (red, **A**,**D**), SV2C-LIR (green, **B**,**E**), and co-localization of peripherin-LIR and SV2C-LIR (yellow, **C**,**F**) in periosteal axons. (**G**–**I**) show images of peripherin-LIR (**G**), SV2C-LIR (**H**), and co-localization ((**I**), same microscope field as (**G**,**H**)). The filled arrowheads mark examples of axons that are double-labeled for SV2C and peripherin. The open arrowheads mark examples of axons that are single-labeled for SV2C only. The arrow in (**I**) indicates the presence of SV2C-LIR axons within peripherin-LIR nerve bundles. The presence of peripherin-LIR confirms that the SV2C-LIR processes are of neuronal origin.

Distribution of SV2C/peripherin-LIR fibers (Figure 6):

Examination of periosteal tissues stained for peripherin- and SV2C-LIR revealed the presence of SV2C-positive fibers in large peripherin-positive axonal bundles, as well as the existence of many individual peripherin-LIR fibers that were SV2C-positive (Figure 6A–F). As in the dura, we could not detect SV2C staining in tissues that were not peripherin-positive (Figure 6C,F,I), again suggesting that SV2C receptors are present in neuronal tissues only. Of all peripherin-LIR fibers, 38% were single-labelled, expressing peripherin-LIR fibers only, and 62% were double-labelled, expressing both peripherin- and SV2C-LIR (Figure 6B,C,E,F,H,I), suggesting the presence of SV2C receptors in a larger percentage of periosteal than dural nerve fibers.

**Figure 7:**
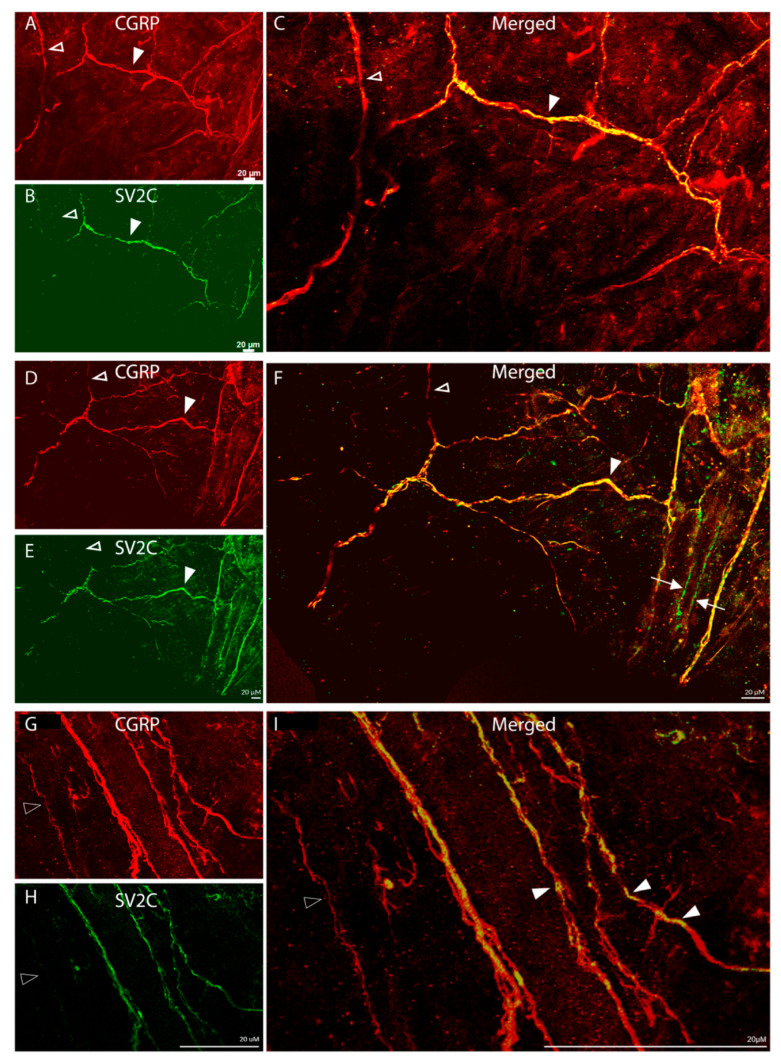
Colocalization of CGRP-LIR and SV2C- LIR in the axons of the periosteum (20× objective). (**A**–**C**), (**D**–**F**), and (**G**–**I**) each show a single microscope field, imaged for either CGRP-LIR (red, **A**,**D**,**G**), SV2C-LIR (green, **B**,**E**,**H**), or colocalization (yellow, **C**,**F**,**I**). The filled arrowheads mark double-labeled axons. The open arrowheads mark single-labeled CGRP-LIR axons. Note the presence of SV2C-LIR in CGRP-LIR axons (yellow), suggesting that some but not all C-fibers express the SV2C receptors (**C**,**F**,**I**). Although uncommon, note that in this tissue (unlike in the dura), SV2C-LIR receptors are distributed in axons that are not CGRP-positive (the arrows in (**F**)), raising the possibility that in the periosteum these receptors may be located in non-C-fiber axons.

Distribution of SV2C/CGRP-LIR fibers (Figure 7):

Fibers showing CGRP-LIR were also seen mainly within large fiber bundles (Figure 7A,D,G). Of all CGRP-LIR fibers, 47% were single-labelled, expressing CGRP-LIR only, and 53% were double-labelled, expressing both CGRP- and SV2C-LIR (Figure 7B,C,E,F,H). As in the dura, SV2C-LIR exhibited a continuous distribution in some axons and a patchy distribution in others (Figure 7I), suggesting that some axons express SV2C receptors throughout their course, whereas others may express these receptors in distinct parts only. Unlike in the dura, a few SV2C-LIR fibers were not CGRP-LIR (Figure 7F), raising the possibility that some SV2C receptors may be expressed by myelinated fibers.

**Figure 8:**
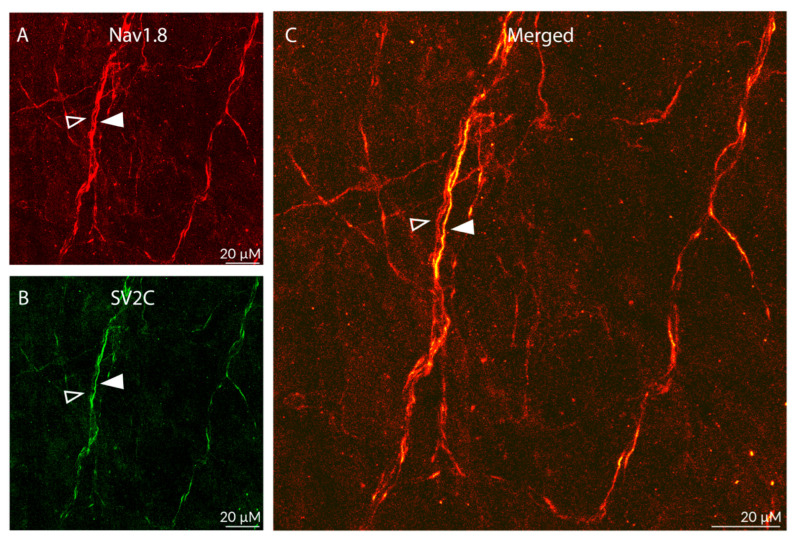
Colocalization of Nav1.8-LIR and SV2C-LIR in axons of the periosteum (20s objective). (**A**–**C**) Images of Nav1.8-LIR (red, **A**), SV2C-LIR (green, **B**), and colocalization (yellow, **C**) in periosteal axons. The filled arrowheads depict a double-labeled axon. The open arrowheads depict a single-labeled Nav1.8-LIR axon. Note the presence of SV2C-LIR in some Nav1.8-LIR axons (yellow), suggesting the presence of SV2C receptors in some but not all sensory C-fibers.

Distribution of SV2C/NaV1.8-LIR fibers (Figure 8):

As expected, the distribution of fibers containing NaV1.8-LIR was far less dense than the distribution of peripherin- or CGRP-LIR fibers (Figure 8A). Axons showing Nav1.8-LIR were also seen in fiber bundles. Of all Nav1.8-LIR fibers, only 32% were single-labelled, expressing NaV1.8-LIR only, while the majority (68%) were also SV2C-LIR (Figure 8B,C), suggesting that some but not all unmyelinated C-class meningeal nociceptors express SV2C receptors. All SV2C-LIR fibers were also NaV1.8-LIR (Figure 8C), suggesting that SV2C receptors are expressed primarily in nociceptors and not in non-nociceptive neurons.

## 3. Discussion

Using single- and double-labeling techniques to map the distribution of axons containing SV2C, the third isoform of the SV2 glycoprotein [28], in mouse dura and periosteum, we describe dense networks of axons containing SV2C- and peripherin-LIR, SV2C- and CGRP-LIR, and SV2C- and NaV1.8-LIR. We interpreted these findings as suggesting that many of the labeled axons are peripheral nerve fibers (peripherin-positive) of sensory and possibly sympathetic/parasympathetic origin (CGRP-positive), and that some of these sensory nerve fibers are nociceptors (NaV1.8-positive). A recent study showing that SV2C is widely co-expressed with CGRP and peripherin in human DRG neurons further supports the presence of SV2C-positive CGRP-positive axons in the meninges [29]. Clinically, these findings demonstrate an abundance of axons containing onabotA receptors in the vicinity of scalp structures commonly injected with onabotA for the treatment of chronic migraine. Dense labeling in the periosteum provides another rationale for the possibility that onabotA injections in this layer of the scalp may be advantageous.

To account for onabotA effects on vesicular trafficking, exocytosis, and stabilization of stored transmitters [30,31,32,33], onabotA must be internalized to synaptic terminals. This internalization is mediated by the binding of the carboxyl terminal segment of the heavy chain to the large intravesicular domain of the synaptic vesicle glycoprotein 2C [27]. Given that (1) for the treatment of chronic migraine onabotA is injected into multiple scalp regions containing pericranial muscles and the tendons that attach them to the periosteum, and (2) of the 3 SV2 isoforms, onabotA has been shown to interact with the large intravesicular domain of SV2C but not SV2A or SV2B [27], the dense presence of SV2C (i.e., the onabotA receptors) in the periosteal axons is fundamental to our understanding of its mechanism of action in migraine prevention [16,17] and the selective attenuation of synaptic transmission in unmyelinated C-fibers [13,15,18].

For many years, it has been evident that the headache phase of migraine depends on activation of trigeminal nociceptors [23] and, in particular, intracranial meningeal nociceptors [23,24,25]. While the current study shows a dense distribution of onabotA receptors in periosteal areas located in the vicinity of the standard 21 injection sites in the scalp, it also shows a dense SV2C distribution in the dura. The presence of SV2C in the dura raises the possibility that onabotA can disrupt synaptic transmission in the dura—if it can somehow reach this intracranial tissue. This possibility calls attention to the need to intensify research into imaging methods (e.g., immunohistochemical labeling, radiolabeling, chemogenetic manipulation) that allow us to reliably trace the location and distribution of the heavy and or light chains of onabotA. In the absence of these techniques, the viability of this possibility cannot be tested.

Peripherin is a 57-kD type III intermediate filament protein that is a specific marker for peripheral neurons [34]. The co-existence of SV2C and peripherin in periosteal and dural axons suggests that this onabotA receptor is present mainly in sensory [35] and potentially sympathetic/parasympathetic [36] peripheral axons. The presence of peripherin in axons of motor neurons [37] can be excluded as such axons do not travel within the dura or periosteum. The presence of SV2C-LIR in axons that are also peripherin-positive (but not in peripherin-negative axons) suggests that SV2C receptors are located mainly, if not exclusively, in neuronal tissue.

To further define the identity of the SV2C-positive axons in the dura and periosteum, we double-labeled them with CGRP. CGRP is a highly potent vasodilator localized primarily in unmyelinated C-fibers of sensory trigeminal ganglion neurons [38], postganglionic sympathetic neurons of the superior and middle cervical ganglia [39], and a small number of postganglionic parasympathetic neurons in the ciliary, otic, sphenopalatine, and submandibular ganglia [40]. The strength of evidence for CGRP’s role in the pathophysiology of migraine [41] and, specifically, its role in provoking migraine in patients [42,43,44], migraine-like behavior in animal models of intracranial pain [45,46], and dilatation of dural and other cranial arteries [47] suggests that the mechanism of action by which onabotA reduces monthly migraine days in CM patients is likely to involve the disruption of synaptic transmission at termination sites of unmyelinated sensory and possibly autonomic C-fibers containing CGRP in the dura and periosteum. The presence of SV2-LIR in approximately half of all CGRP-positive axons and the abundance of sensory and autonomic axons in the dura and periosteum raise several possibilities. The first is that dural and periosteal axons containing CGRP and SV2C are sensory but not autonomic, the second is that they are autonomic but not sensory, and the third is that not all unmyelinated C-fibers originating from the autonomic ganglia and trigeminal ganglion contain SV2C and thus can be affected by onabotA therapy.

As the presence of CGRP in SV2C-positive axons cannot distinguish with 100% accuracy between nociceptors and autonomic axons, we used Nav1.8 to identify the presence of SV2C receptors in nociceptors. Nav1.8 is a tetrodotoxin-resistant voltage-gated channel that plays a significant role in the initiation and persistence of chronic pain [48]. It is expressed almost exclusively in unmyelinated, small-diameter primary sensory neurons that play a major role in the transmission of pain-related signals in dorsal root and trigeminal ganglia [49,50,51,52]. The presence of SV2C-LIR in the majority of NaV1.8-positive axons suggests that SV2C receptors are found in many C-fiber nociceptors in the dura and periosteum. Future studies are warranted on how the level of activation and sensitization of sensory axons impact receptor expression and distribution, and how exposure to onabotA alters cleaved SNAP25 expression and distribution, as this may allow us to better understand the mechanism of action.

The present data show that the distribution of onabotA receptors in areas involved in the generation of the headache phase of migraine is wider than previously thought and that the internalization of onabotA can be selective for or show distinct preference for some but not all groups of sensory neurons (e.g., trigeminal nociceptors) and, potentially, sympathetic neurons. These findings may help explain onabotA’s ability to inhibit C- but not Aδ or Aβ sensory neurons and prevent chronic migraine without causing numbness or loss of non-nociceptive mechanical and thermal sensations. Therapeutically, the dense distribution of SV2C receptors in the periosteum raises the possibility that onabotA injections near this bony structure may have for some patients an improved therapeutic outcome than the current practice of injecting this neurotoxin intramuscularly. Further studies will be needed to compare clinical efficacy of injections made near periosteal vs. muscular tissues.

## 4. Materials and Methods

### 4.1. Animals

Fifteen C57BL/6 wild-type mice weighing 20–30 g were used in this study. Experiments were conducted in accordance with NIH guidelines and approved by the Institutional Animal Care and Use Committee at Harvard Medical School and Beth Israel Deaconess Medical Center (protocol number 053-20-23; approved 22 March 2024). Mice were housed in a controlled environment (22 °C RT; 12 h light/dark cycle), 2 to 5 per cage, with ad libitum access to food and water.

### 4.2. Tissue Collection and Processing

Mice were deeply anesthetized using intraperitoneal injection of urethane (1.5 g/kg) and atropine (0.15 mg/kg). Transcardial perfusion was performed using a brief (1 min) phosphate-buffered saline (PBS) infusion that was followed by 4% paraformaldehyde (PFA) for 4 min. This protocol minimizes perfusion-related hypoxic stress on neurons and non-specific immunohistochemistry artifacts [53]. The skull containing the dural meninges was then post-fixed for 10 min in 4% PFA at room temperature. Dura and periosteum were extracted from the skull and preserved in PBS.

### 4.3. Immunohistochemistry

Free-floating, whole-mount dura and periosteum were pre-incubated at room temperature in PBS containing 10% donkey serum albumin and 0.5% Tween for 1 h, followed by incubation for 48 h with primary antibodies against SV2C (rabbit anti-SV2C; 1:300; Abcam, Cambridge, UK; RRID:AB_33892-1001) and/or peripherin (chicken anti-peripherin; 1/300; Abcam, RRID:AB_39374) and/or CGRP (goat anti-CGRP; 1:500; Abcam, catalog #Ab36001; RRID:AB_725807) and/or NaV1.8/SCN10A (mouse anti-NaV1.8/SCN10A; 1:400; Abcam, catalog #S134; RRID:AB_93616-1001). The SV2C antibody we used is a rabbit polyclonal, and due to the homology of the antigen used to generate the antibody (https://doc.abcam.com/datasheets/inactive/ab33892/en-us/sv2c-antibody-ab33892.pdf, accessed on 3 March 2024), it may cross-hybridize with other SV2 family members (e.g., SV2A and B) whose expression in human and TG cells is far lower than the expression of SV2c.

The tissues were rinsed in PBS and incubated for 2 h at room temperature with the corresponding fluorescent secondary antibody (1:300; AlexaFluor 488 or 555; ThermoFisher Scientific, Waltham, MA, USA, catalog #A-11008 (RRID: AB_143165) and catalog #A-11037 (RRID:AB_2534095)) against the IgGs of the species in which the primary antibody was raised. All immunolabeled tissues were mounted in 1:1 glycerol:PBS medium containing 12.5 mg/mL sodium azide and examined by epifluorescence and laser-scanning confocal microscopy.

### 4.4. Digital Imaging of Dura and Periosteum

Image acquisition of labeled axons in the dural tissues covering the cerebral cortex along the temporal aspect of the brain and in the periosteal tissue of the calvaria was done at a resolution of 1024 × 1024 pixels, with a scan rate of 8–10 μs/pixel, with or without zoom. To reduce excitation and emission crosstalk, images were acquired sequentially, line by line. Exposure settings that minimized oversaturated pixels in the final images were used. AlexaFluor-555 and AlexaFluor-488 were excited using 559 and 488–515 nm laser diodes lines, respectively. Photomicrographs of double-labeling were obtained by superimposition of green and red images.

**Figure 9:**
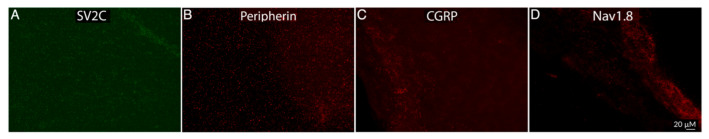
Control and specificity of staining in dural whole-mounts. The images show sections lacking primary antibodies for SV2C (**A**), peripherin (**B**), CGRP (**C**), and Nav1.8 (**D**) (20× objective).

Using ImageJ (ver.1.54), the images were adjusted for color and brightness for presentation purposes and a scale bar was added. Any adjustment was applied uniformly across all images for each antibody. Staining was defined as detectable when it could be visually discerned above background compared to controls that were not exposed to primary antibody under identical imaging conditions. For each experiment, one slide per condition was not exposed to primary antibody as a negative control (Figure 9). The image panels were prepared using Adobe Photoshop.

## Data Availability

The original contributions presented in this study are included in the article. Further inquiries can be directed to the corresponding author(s). Please note that original processed tissue cannot be shared due to fading of fluorescent signals over time.
